# Electronic Nose Breathprints Are Independent of Acute Changes in Airway Caliber in Asthma

**DOI:** 10.3390/s101009127

**Published:** 2010-10-12

**Authors:** Zsofia Lazar, Niki Fens, Jan van der Maten, Marc P. van der Schee, Ariane H. Wagener, Selma B. de Nijs, Erica Dijkers, Peter J. Sterk

**Affiliations:** 1 Department of Pulmonology, Semmelweis University, Diósárok u. 1/c, 1125 Budapest, Hungary; 2 Department of Respiratory Medicine, Academic Medical Centre, University of Amsterdam, Meibergdreef 9, 1105 AZ Amsterdam, The Netherlands; E-Mails: n.fens@amc.uva.nl (N.F.); m.p.vanderschee@amc.uva.nl (M.P.D.S.); a.h.wagener@amc.uva.nl (A.H.W); s.b.denijs@amc.uva.nl (S.B.D.N.); e.dijkers@amc.uva.nl (E.D.); p.j.sterk@amc.uva.nl (P.J.S.); 3 Department of Pulmonology, Medical Centre Leeuwarden, PO Box 888, 8901 BR Leeuwarden, The Netherlands; E-Mail: j.maten@znb.nl

**Keywords:** volatile organic compounds, exhaled breathprint, electronic nose, pattern recognition, airway caliber, bronchial asthma, bronchial provocation

## Abstract

Molecular profiling of exhaled volatile organic compounds (VOC) by electronic nose technology provides breathprints that discriminate between patients with different inflammatory airway diseases, such as asthma and COPD. However, it is unknown whether this is determined by differences in airway caliber. We hypothesized that breathprints obtained by electronic nose are independent of acute changes in airway caliber in asthma. Ten patients with stable asthma underwent methacholine provocation (Visit 1) and sham challenge with isotonic saline (Visit 2). At Visit 1, exhaled air was repetitively collected pre-challenge, after reaching the provocative concentration (PC_20_) causing 20% fall in forced expiratory volume in 1 second (FEV_1_) and after subsequent salbutamol inhalation. At Visit 2, breath was collected pre-challenge, post-saline and post-salbutamol. At each occasion, an expiratory vital capacity was collected after 5 min of tidal breathing through an inspiratory VOC-filter in a Tedlar bag and sampled by electronic nose (Cyranose 320). Breathprints were analyzed with principal component analysis and individual factors were compared with mixed model analysis followed by pairwise comparisons. Inhalation of methacholine led to a 30.8 ± 3.3% fall in FEV_1_ and was followed by a significant change in breathprint (p = 0.04). Saline inhalation did not induce a significant change in FEV_1_, but altered the breathprint (p = 0.01). However, the breathprint obtained after the methacholine provocation was not significantly different from that after saline challenge (p = 0.27). The molecular profile of exhaled air in patients with asthma is altered by nebulized aerosols, but is not affected by acute changes in airway caliber. Our data demonstrate that breathprints by electronic nose are not confounded by the level of airway obstruction.

## 1. Introduction

Asthma is a chronic inflammatory disorder of the airways characterized by recurrent episodes of wheezing and chest tightness that are associated with variable airway obstruction. Asthma diagnosis is established based on symptoms, measurement of lung function and assessment of airway responsiveness [1]. In addition, the associated airway inflammation can be evaluated by validated non-invasive techniques such as sputum eosinophil counts [2] and exhaled nitric oxide level (NO) [3], which have shown to be useful in monitoring asthma.

Exhaled air is a mixture of thousands of volatile organic compounds (VOCs) [4], which are generated via metabolic pathways that may be altered by lung diseases. Identification and quantification of individual VOCs require laboratory methodologies employing gas chromatography coupled to mass spectrometry (GC-MS). Indeed, panels of VOCs enable the distinction of smoking and non-smoking healthy subjects [5], and can identify lung cancer with sufficient sensitivity and specificity [6]. Less laborious peak pattern analysis after spectrometry without direct VOC characterization has recently been successful in recognizing lung cancer patients [7]. Nevertheless, the procedure still requires skilled personnel and advanced technical facilities, limiting potential widespread medical applicability.

Electronic nose (eNose) technology provides a cost-effective on-site alternative for breath analysis. eNoses exploit arrays of broadly cross-reactive sensors responding to a variety of VOCs in a highly sensitive and reversible manner within a short response time [8,9]. eNoses generate a molecular profile of the VOC mixture in exhaled breath also called the breathprint, and allow analysis by pattern recognition algorithms for discrimination between individual breathprints without identifying the individual analytes. An eNose with carbon black polymer was able to predict pneumonia in ventilated patients [10], distinguish patients with lung cancer from COPD [11], discriminate asthma patients from controls [12] and from COPD patients [13]. In addition, an array of sensors based on gold nanoparticles was able to distinguish lung cancer patients from controls [14] and an electronic nose with quartz microbalance gas sensors could also discriminate asthma patients from healthy subjects [15]. Due to easy sampling procedures, portability and relatively low cost, eNose technology might be useful in medical decision making [16], which requires strict procedures to assess diagnostic accuracy (www.stard-statement.org).

Although the discriminative potential by eNoses is encouraging, patient-related and methodological issues have been raised concerning breath collection and analysis [15,17]. Expiratory volume and flow need to be standardized [12], but it is unknown whether the breathprints can be modulated by airway caliber. For exhaled NO it has been reported that airway narrowing leads to a reduction of exhaled NO in asthma [18,19], although this could not be confirmed in another study [20]. A salbutamol-induced acute increase in airway caliber may elevate exhaled NO level [21], although this is not a consistent finding [19,22]. Thus, it cannot be excluded that other exhaled components, such as VOCs, are also affected by acute changes in airway caliber. If so, this might complicate the interpretation of breathprints in general and particularly in asthma.

Therefore, the null-hypothesis of this study was that breathprints assessed by electronic nose technology are not affected by airway caliber. To investigate this we recorded breathprints before and at acute changes in airway caliber during methacholine provocation in asthmatic patients. A control challenge with nebulised isotonic saline was performed to examine any confounding effects of the challenge procedure on the breathprint. Finally, we assessed the between-day variability in asthmatics by comparing pre-challenge baseline breathprints.

## 2. Experimental Section

### 2.1. Study design

The effect of bronchoconstriction on exhaled breathprints was examined in a cross-over study performed on asthma patients (n = 10) attending two visits with a mean time between visits of 7.5 days (range 4–14 days) at a similar time of the day (± 2 h). At Visit 1, methacholine (MCh) provocation and at Visit 2, a sham challenge with isotonic saline was performed. At Visit 1 exhaled breath was collected before MCh provocation (baseline), when at least a 20% drop in FEV_1_ was achieved (post-methacholine) and after salbutamol inhalation (post-salbutamol). At Visit 2, exhaled air was collected before sham challenge (baseline), after the last inhalation of saline (post-saline) and after salbutamol inhalation (post-salbutamol). Subjects were not blinded to the procedures and visits were not randomized because of the need to match the number of inhaled doses between the visits.

The effect of methodological drifts in eNose signals was investigated in a control study performed in 10 volunteers (seven healthy, three asthmatics: see below). Breath sampling was performed three times following the same course as during the challenge procedures in the main study but without any intervention (0 min, 60 min and 90 min). Subjects did not eat and drank only water during and 3 hours prior to that period.

### 2.2. Subjects

Ten adult patients with previously diagnosed asthma agreed to participate in the main study. The patients were never-smokers and had episodic chest tightness or wheezing with a pre-bronchodilator FEV_1_ >65% predicted and documented airway responsiveness (PC_20_ methacholine <8 mg/mL) or reversibility in FEV_1_ predicted >12% after 400 μg inhaled salbutamol as established within 12 months prior to the study. Subjects with concurrent pulmonary disorders, diabetes mellitus, hypo- or hyper-thyroidism, severe cardiovascular disease, renal insufficiency, present cancer or cancer in the past 5 years, oral corticosteroid use, present parodontitis or recent dental treatment were excluded. Patients on inhaled medications other than short-acting or long-acting β_2_-agonists and/or inhaled steroids or those who had a history of upper or lower respiratory tract infection in the four weeks before the measurements were excluded from the study.

Ten volunteers, including seven healthy non-smoking subjects with no previous history of airway diseases or other chronic diseases, and three asthmatics but otherwise healthy patients, without an upper or lower respiratory tract infection in the four weeks before the measurements, were recruited to participate in the control study for eNose drift analysis. The protocol was approved by the local medical ethics committee. Written informed consent was obtained from all subjects.

### 2.3. Methacholine and sham challenges

MCh challenge test was performed according to the 2-min tidal breathing method [23]. The patients inhaled methacholine with tidal breathing for 2 min, and FEV_1_ was recorded 30 s and 90 s after the exposure (MasterscreenPneumo; Jaeger; Würzburg, Germany). Doubling doses of methacholine bromide ranging from 0.04 to 19.6 mg/mL were applied in 5-min intervals until PC_20_ was achieved. PC_20_ was calculated with linear interpolation. Subsequently, the patients inhaled 400 μg salbutamol per metered dose inhaler with a spacer, and after 10 min FEV_1_ was measured. FEV_1_ was considered to be restored if higher than 90% of baseline.

Sham challenge with 0.9% isotonic saline solution (154 mM NaCl) was performed in identical fashion with identical numbers of aerosol inhalations and spirometric maneuvers as performed when achieving PC_20_.

Patients withheld long-acting β2 agonists and antihistamines for 48 hours, and short-acting β2 agonists and inhaled corticosteroids >8 hours before both challenge tests.

### 2.4. Exhaled breath collection and electronic nose sampling

To reduce possible confounding effects, patients were asked not to eat and drink anything but water 3 h prior to breath collection and refrain from caffeine-containing beverages and peppermint exposure for 6 hours before the visits. Breath collection and sampling were performed using the 5-min tidal washin method [12]. Briefly, patients inhaled VOC-filtered air (A2, North Safety, Middelburg, Netherlands) and exhaled via a silica reservoir in a 2-way non-rebreathing valve (Hans Rudolph 2700, Hans Rudolph, Kansas City, MI, US). Following 5 min of equilibration with tidal breathing, patients performed a maximal inspiratory capacity maneuver and the full expiratory capacity volume was collected into a 10-L Tedlar bag. Bags were sampled within ten minutes followed by the parallel sampling of another Tedlar bag containing VOC-filtered room air as a reference.

Exhaled breath samples were analyzed at room temperature by the same handheld electronic nose (Cyranose 320; Smiths Detection, Pasadena, CA, US) with an array of 32 carbon black polymer sensors [8,9]. VOCs binding to a sensor cause a change in the electrical resistance of the sensor. The raw data of a breathprint compose of 32 values each corresponding to the relative change in electrical resistance of a sensor. According to the manufacturer’s instruction, the first measurement was disregarded at each session (first sniff effect).

### 2.5. Data analysis

Sensor data of the eNose were processed through Savitzky-Golay filtering and baseline correction. Offline analysis of raw data was performed using SPSS software (version 16.0). Principle component analysis was used to redistribute the variance of the original 32 sensors into a set of factors (four factors captured 95.6% of the variance within the data set in the main study and 97.3% of the variance at the control visit). The two factors (denoted as Factor 2 and Factor 3) which showed a significant response to methacholine and/or salbutamol inhalation were selected for further analysis and are being referred to as “the breathprint”.

A mixed model analysis followed by pairwise comparisons on factors was used to evaluate any change in breathprints when recorded repetitively during the visits. To compare the changes (deltas) in breathprints caused by nebulisation with methacholine and isotonic saline, corresponding baseline factors were subtracted from post-methacholine or post-saline factors, and these changes were compared with paired t-tests for each factor. To assess between-day variability of a breathprint, baseline factors at visits were compared with paired t-tests.

There are no previous data for calculation of the statistical power of studies with serial eNose measurements. However, previous parallel studies have shown adequate power at similar samples sizes [11,12]. Therefore, the current sample size was considered to be adequate for within-subject analysis.

FEV_1_ values recorded within a visit were compared with mixed model linear followed by pairwise comparison, the percentage change in FEV_1_ at the visits and baseline FEV_1_ values between visits were analyzed with paired t-tests. Data were normally distributed (Kolmogorov-Smirnov test) and are expressed as mean ± SEM in the figures and as mean ± SD in the table and in the text. The level of significance was considered as p < 0.05.

## 3. Results and Discussion

### 3.1. Study population

Patient characteristics and baseline lung function parameters are presented in Table 1. The population was well-characterized, but we acknowledge that the number of subjects in this study was limited. Even though data for formal power calculations of serial eNose measurements are lacking, similar numbers of patients were successfully used in previous eNose studies [11,12] as well as in former studies on the effects of airway caliber on exhaled NO [18,20] based on adequate power calculations.

### 3.2. Methacholine and sham challenges modify breathprints

To investigate the effect of airway caliber on exhaled breathprints, we chose a controlled, cross-over challenge model with methacholine as well as sham (saline) provocation. This allowed the distinction in outcomes as produced by methacholine and inhaled aerosols or the procedure as such.

**Figure 1.**
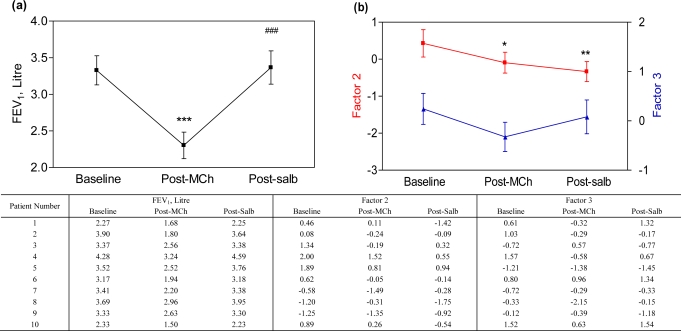
**(a)** FEV_1_ measurements at baseline, after methacholine (Post-MCh) inhalation and post-salbutamol (Post-salb). **(b)** Breathprints at baseline, after methacholine inhalation and post-salbutamol are presented by plotting Factor 2 (red line) and 3 (blue line). *p < 0.05, **p < 0.01, *** p < 0.001 *vs.* baseline; ^###^p < 0.001 *vs.* post-methacholine. The data are shown in the table below the figure.

As expected, methacholine inhalation induced significant bronchoconstriction (baseline FEV_1_ 3.33 ± 0.63 L *vs.* 2.30 ± 0.57 L post-methacholine, p < 0.001), and subsequently, airway caliber was restored by salbutamol inhalation [post-salbutamol FEV_1_ 3.37 ± 0.72 L, p < 0.001, Figure 1(a)]. Breathprints, as demonstrated by Factor 2, were significantly changed after methacholine (p = 0.04) and also post-salbutamol (p = 0.006) when compared to the breathprints at baseline; however, the breathprints after salbutamol were not significantly different from the ones after methacholine [p = 0.34, Figure 1(b)].

**Figure 2.**
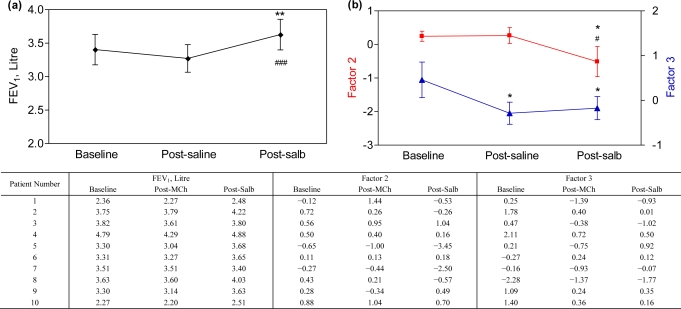
**(a)** FEV_1_ measurements at baseline, after saline inhalation and post-salbutamol (post-salb). **(b)** Breathprints at baseline, after saline inhalation and post-salbutamol are presented by plotting Factor 2 (red line) and 3 (blue line). *p < 0.05, **p < 0.01 *vs.* baseline; ^#^p < 0.05, ^###^p < 0.001 *vs.* post-saline. The data are shown in the table below the figure.

Inhalation of isotonic saline did not change FEV_1_ (baseline 3.40 ± 0.72 L *vs.* post-saline FEV_1_ 3.27 ± 0.65 L, p = 0.13), but FEV_1_ increased to 3.63 ± 0.73 L after salbutamol inhalation [post-salbutamol *vs.* baseline and post-saline: p = 0.008 and p < 0.001, Figure 2(a)]. Breathprints were significantly changed post-saline (Factor 3: p = 0.01) and post-salbutamol (Factor 2: p = 0.02, Factor 3: p = 0.03) when compared to the breathprints at baseline. The post-salbutamol breathprint was also altered as compared to post-saline [Factor 2: p = 0.02, Figure 2(b)].

Our primary aim was to study the effect of bronchoconstriction on breathprints, but we additionally analyzed the effect of acute bronchodilation induced by salbutamol after the provocation test. Our data show that an acute and marked increase in airway caliber after methacholine challenge does not modify exhaled breathprints. Nevertheless, we cannot exclude any carry-over effect of inhaled methacholine on the post-salbutamol breathprints. Notably, the small but significant increase in airway caliber by salbutamol following sham provocation unexpectedly altered the breathprint. We cannot explain this observation and its inconsistency with regard to giving salbutamol after methacholine challenge. It may suggest that salbutamol inhalation in the absence of methacholine challenge as such provided a signal on the breathprint similar to the sham challenge with inhaled saline.

As temporal drift in polymer sensors due to incomplete desorption of molecules from sensor surfaces has been reported [24], a control study was done, in which non-smoking volunteers followed the same course of three repeated eNose analyses without any intervention. We found no significant temporal change in the four factors analyzed (0 min *vs.* 60 min *vs.* 90 min; Factor 1: 0.12 ± 0.94 *vs.* −0.50 ± 0.97 *vs.* 0.38 ± 0.97, p = 1.00; Factor 2: 0.61 ± 0.91 *vs.* −0.07 ± 0.99 *vs.* −0.54 ± 0.82, p = 0.66; Factor 3: 0.52 ± 1.22 *vs.* −0.17 ± 0.84 *vs.* −0.34 ± 0.75, p = 0.16; Factor 4: −0.23 ± 1.22 *vs.* 0.01 ± 0.86 *vs.* 0.23 ± 0.93, p = 1.00). This suggests that the change in breathprints observed during the challenge procedures is not the result of sensor drifts in the eNose.

### 3.3. Changes in breathprints are not related to bronchoconstriction

**Figure 3.**
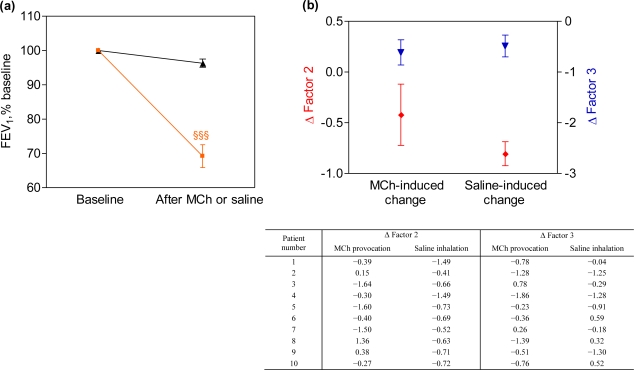
**(a)** Changes in FEV_1_ after the inhalation of methacholine (MCh; orange line) or isotonic saline (black line). **(b)** Changes in breathprints induced by methacholine or saline inhalation as the change (delta) in Factor 2 (red line) and 3 (blue line); for all deltas: p > 0.05. ^§§§^p < 0.001 post-methacholine *vs.* post-saline.

As expected, the decrease in FEV_1_ by MCh provocation was more marked than after saline [−30.8 ± 10.5% *vs.* −3.7 ± 3.8%, p < 0.001, Figure 3(a)]. However, there was no significant difference between the changes (deltas) in breathprints as induced by MCh provocation and sham challenge [delta Factor 2: p = 0.27, delta Factor 3: p = 0.66, Figure 3(b)].

How can these results be explained? The similar changes in breathprint after challenges with methacholine and isotonic saline indicate that alterations in breathprint might be due to the nebulisation procedure itself rather than to the change in airway caliber. We used a previously validated sampling technique with filtering of VOCs in inspired air and drying of exhaled air [12] in order to minimize the influence of humidity, expiratory flow rate and environmental VOCs on the exhaled VOC mixture. Although exhaled air is fully saturated, even under physiological conditions, we cannot exclude an effect of humidity on the breathprints after inhaling nebulized solutions. The current findings suggest that the drying step in the breath collection procedure may need to be optimized when used after aqueous nebulization. This issue might be particularly relevant in future studies where patients are subjected to nebulized therapies. An alternative explanation is that isotonic saline (alone or as a diluent for MCh), having a chloride concentration of 154 mM that is higher than that of the epithelial lining fluid (115 mM), induces metabolic changes in the bronchial epithelium [25]. If so, this could affect exhaled VOC profile thereby modifying exhaled breathprints. This possibility might be examined by local bronchoscopic fluid installation and air sampling.

### 3.4. Breathprints do not show between-day variability in asthma patients

**Figure 4.**
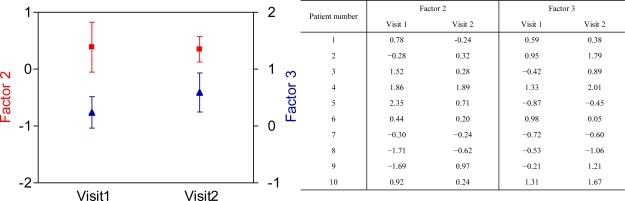
Baseline breathprints are unchanged in asthmatic patients. Pre-challenge baseline breathprints at the two visits are shown by plotting Factor 2 (red line) and Factor 3 (blue line); for all factors: p > 0.05. The attached table shows the data.

Baseline FEV_1_ values were similar between the two visits (Visit 1: 3.33 ± 0.63 L *vs.* Visit 2: 3.40 ± 0.72 L, p = 0.34), and neither was there a significant difference in baseline breathprints (Factor 2: p = 0.92, Factor 3: p = 0.18, Figure 4). These findings demonstrate that breathprints do not show between-day variability in non-smoking asthma patients. This extends a previous study reporting no differences in breathprints in healthy smoking and non-smoking control subjects when recorded at two separate days [13]. As variability of breathprints is an important issue in the standardization of breath analysis, further large-scale studies should confirm these findings.

## 4. Conclusions

This study shows that an acute decrease in airway caliber *per se* is not associated with an altered breath molecular profile in asthma as measured by an electronic nose. This suggests that eNose assessment in asthma does not require correction for the degree of airway obstruction. We also demonstrate that inhaled nebulized aerosols can change breathprints irrespective of the change in airway caliber. Finally, breathprints by an eNose do not show significant between-day changes in stable asthma.

These findings imply that the previously observed discrimination of patients with asthma, COPD and controls by breathprint analysis [12,13,15] is due to the differences in underlying inflammation or disease activity rather than the level of airway obstruction. The clinical implication of this study is that monitoring of patients with asthma with repeated eNose assessments can be performed, regardless of acute changes in airway caliber. Exhaled molecular profiles may still vary with the degree of airway obstruction if the latter goes along with changes in airway inflammation. This strengthens the applicability of eNose technology in the future monitoring of diseases with variable airway obstruction and facilitates further studies on the validation of eNose monitoring in patients with asthma and COPD.

## Figures and Tables

**Table 1.** Patient characteristics. Spirometric data are pre-challenge baseline values at Visit 2. FVC: forced vital capacity, FEV_1_: forced expiratory volume in 1 s, ICS: inhaled corticosteroids, Bud Eq: budesonide equivalent, PC_20_: provocative concentration of methacholine causing 20% fall in FEV_1_, MCh: methacholine, Post-salb: post-salbutamol inhalation.

**Patient Number**	**Sex**	**Age, years**	**Daily ICS (bud eq, μg)**	**Baseline FVC, % predicted**	**Baseline FEV_1_, % predicted**	**Baseline FEV_1_/FVC**	**PC_20_ MCh, mg/mL**	**FEV_1_ change at Visit1^&^**		**FEV_1_ change at Visit2^&^**	

								**PC_20_ MCh**	**Post-salb**	**Post-saline**	**Post-salb**
1	F	33	400	102	89	0.76	12.93	−26	0	−4	+6
2	F	27	800	119	104	0.77	5.18	−54	−7	−1	+13
3	M	29	400	99	82	0.71	0.22	−24	0	−6	0
4	M	35	200	129	106	0.67	6.93	−24	+7	−11	+2
5	M	47	200	128	84	0.53	0.76	−28	+7	−8	+11
6	F	33	0	108	98	0.80	1.33	−39	0	−1	+10
7	F	23	200	103	101	0.85	3.13	−36	−1	0	−3
8	F	30	0	106	92	0.85	0.31	−20	+7	−1	+11
9	M	30	800	88	72	0.67	0.58	−21	−1	−4	+11
10	F	45	200	87	79	0.78	1.39	−26	−4	−3	+11

		33 ± 8	400 ± 262^#^	107 ± 15	91 ± 12	0.74 ± 0.01	1.55 (0.59–4.10)	−31 ± 11	1 ± 5	−4 ± 4	7 ± 6

& Change in percentage compared to corresponding baseline FEV_1_ values.

# Only ICS users considered. Data are expressed as mean ± SD or geometric mean (95% confidence interval).
